# Effect of Heat Treatment on Crevice Corrosion Behavior of 304 Stainless Steel Clad Plate in Seawater Environment

**DOI:** 10.3390/ma16113952

**Published:** 2023-05-25

**Authors:** Pengwei Hang, Boshen Zhao, Jiaming Zhou, Yi Ding

**Affiliations:** 1 College of Materials Science and Engineering, Nanjing Tech University, Nanjing 210009, China; 2 Shangi Institute for Advanced Materials Co., Ltd., Nanjing 210000, China

**Keywords:** stainless steel clad plate, quenching and tempering treatment, crevice corrosion, electrochemical test

## Abstract

With the application of stainless steel clad plate (SSCP)-enlarging in the marine engineering field, awareness of the consequences of heat treatment on ameliorating microstructure and mechanical properties in stainless steel (SS)/carbon steel (CS) joints is being raised. However, carbide diffusion from a CS substrate to SS cladding may damage the corrosion resistance during inappropriate heating. In this paper, the corrosion behavior of a hot rolling-produced stainless steel clad plate (SSCP) after quenching and tempering (Q-T) treatment, especially crevice corrosion, was studied by electrochemical and morphological methods, such as cyclic potentiodynamic polarization (CPP), confocal laser scanning microscope (CLSM) and scanning electron microscopy (SEM). Q-T treatment led to more significance in carbon atoms diffusion and carbide precipitation, which made the passive film of the SS cladding surface on the SSCP unstable. Subsequently, a device for measuring the crevice corrosion performance of SS cladding was designed; the Q-T-treated cladding showed lower re-passivation potential (−585 mV) during CPP when compared to as-rolled (−522 mV), with the maximum corrosion depth ranging from 70.1 μm to 150.2 μm. In addition, the processing of crevice corrosion on SS cladding could be divided into three parts, including the initiation, propagation and development stages, which were driven by the interactions between corrosive media and carbides. The generation and growth mechanism of corrosive pits in crevices were revealed.

## 1. Introduction

Nowadays, stainless steel (SS)/carbon steel (CS) bimetallic structures also known as stainless steel clad plates (SSCP) are gaining wider application in marine engineering, petrochemical and nuclear engineering [1], due to its high strength of base carbon steel and corrosion resistance of cladding stainless steel. With the development of the SSCP manufacturing process, more stainless steel/carbon steel bimetallic profiles have been produced, such as pipes, bars and fasteners [2,3,4]. However, as a result of tight assembly, some bimetallic portions have the risk of localized corrosion problems, such as crevice corrosion [5,6,7] between SSCP bolts and SS nuts.

The fact that applied stress, heat treatment, corrosive medium, crevice size, etc. damage the stability of austenitic stainless steel crevice areas has been revealed. Zhu et al. [8] proved the passive film of 304 SS creviced degraded under applied stress in 3.5 wt. % NaCl solution. The tensile stress increases the probability of metastable pits changing into stable pits, thus promoting the development of crevice corrosion. Calabokis et al. [9] found low-temperature plasma-nitrided UNS S32750 at 400 °C for 4 h could improve crevice corrosion resistance, and the alloying Ni element in the nitrided layer played an advantageous role in the propagation and crevice corrosion re-passivation processes. Machuca et al. [10] believed crevice corrosion could be promoted in oxygen-rich environments, the existence of O_2_ enhanced the cathodic reaction process outside the crevice. Yeh et al. [11] proved 304L SS was prone to crevice corrosion when operating in a saline environment with deposited dust. E. Shojaei et al. [12] found that 316L SS was sensitive to crevice corrosion in 3.5 wt. % NaCl solution as the crevice width decreased, because the incubation period of crevice corrosion was shortened. Kim et al. [13] found the crevice corrosion behavior also affected by the crevice-former, such as metal, polytetrafluoroethylene (PTFE) and ethylene propylene diene monomer (EPDM).

However, unlike single austenitic stainless steel, the corrosion behavior of SSCP with the crevice-former may be affected by more complex factors. During common hot-rolling bonding and the post-heat treatment process, the interdiffusion of alloy elements near the SS/CS joint might modify the microstructure and corrosion resistance of surface cladding. Wu et al. [14] proved by increasing the carbon content of the base from 0.06 to 0.2% that carbon-chromide precipitation generated along the austenite grain boundary because of excessive carbon atoms diffusion in austenitic cladding, which may damage the passive film of the surface. Subsequently, the diffusion of carbon atoms into SS cladding was accelerated, resulting in a wider carburized zone at 700 °C from 2 to 8 h. Meanwhile, the microstructure changed from discontinuous granular to continuous layered, and the corrosion resistance decreased, which was also detected by Li et al. [15]. Xiao et al. [16] believed the addition of a Ni interlayer among the SS cladding and CS base was so effective in preventing element diffusion that SS cladding had an excellent corrosion resistance. In fact, the behavior of carbon element diffusion from the CS side to SS side enhanced the interface bonding shear strength but caused low fracture toughness and corrosion resistance [17]. Interestingly, Liu et al. [18] noticed that both the interfacial bonding strength and corrosion resistance could be improved by quenching the temperature raised from 850 to 1150 °C. Ordinarily, the quenching and tempering (Q-T) heat treatment is employed to improve the comprehensive mechanical properties of medium carbon steel substrates [19]. Some attempts have also been made in the SSCP. For instance, Hyojin Song et al. [20] tried improving the corrosion resistance of cladding S32750 and the mechanical properties of base EH40 in the SSCP after a water-quenching treatment at 1080 °C for 1 h and air cooling after the treatment at 550 °C for 1.5 h.

Currently, the research is focused on the crevice corrosion behavior of single austenitic stainless steel, and the research on the SSCP is mainly focused on the effect of heat treatment on its performance. There is blank research on the effect of heat treatment on crevice corrosion of the SSCP. For quenched and tempered steel, Q-T treatment can improve its comprehensive mechanical properties, but for SS, Q-T treatment inevitably reduces the surface corrosion resistance.

Hence, when the SSCP profile is assembled with the crevice-former, the effect of Q-T treatment on microstructure evolution, element diffusion and the crevice corrosion mechanism needs more concern. In this work, the crevice corrosion behavior of the SSCP after Q-T treatment is carried out by morphology and electrochemical analysis. Meanwhile, the effect of carbon atoms diffusion and carbide precipitation of SS cladding on the corrosion behavior in the SSCP is systematically clarified. Finally, the corrosion mechanism of the SSCP with the crevice-former is established. It is expected that this work could enhance the understanding of the crevice corrosion mechanism of the SSCP after Q-T treatment.

## 2. Materials and Methods

### 2.1. Materials and Heat Treatments

The original SSCP was produced by hot rolling at 1150 °C with 45 steel and 304 SS. Chemical compositions of the raw materials are listed in Table 1. The thickness of the SSCP is 5 mm; among which, the thickness of SS cladding is 0.5 mm. As-hot-rolled 304/45 SSCP was subsequently modified by quenching and tempering heat treatment. Detailed heat treatment parameters and corresponding abbreviations of the specimens are exhibited in Table 2.

### 2.2. Crevice Preparation Procedure

**Figure 1:**
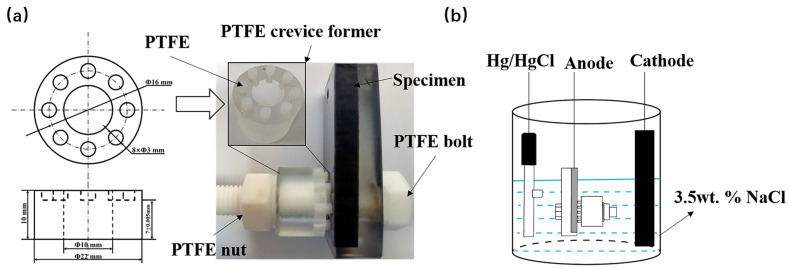
Schematic diagram of (**a**) the assembly for the crevice corrosion test and (**b**) the three-electrode test cell.

A modified crevice assembly for crevice corrosion testing was used by ASTM G48 and G78 [21]. The specimens which dimensions were 35 mm × 25 mm × 5 mm with a 10 mm diameter hole were cut from the SSCP. The modified mold presented in Figure 1a was made of polymethylmethacrylate (PMMA). Eight cylinders 3 mm in diameter made of PTFE were inserted into eight holes of the mold and firmly glued by using a modified acrylate adhesive. A heat shrinking tube was used to insulate the PTFE bolt to prevent an electrical connection between the PTFE bolt and specimen. The connection was done by PTFE nuts, and the torque value was adjusted to vary the crevice width. In this study, the torque value was 3N·M.

### 2.3. Corrosion Performance

Electrochemical tests were performed with a CHI760E electrochemical workstation in a conventional three-electrode cell, which was composed of a reference electrode (saturated calomel electrode), a counter electrode (graphite) and a working electrode (specimen assembly) in Figure 1b. All specimens and crevice-formers were abraded with wet 600 grit SiC paper and degreased with acetone, followed by air-drying. To ensure the stability of the solution, the 1 h open circuit potential (OCP) was required prior to all tests. DL-EPR tests were carried out in 0.5 M H_2_SO_4_ solution + 0.01 M KSCN at room temperature. During the cyclic potentiodynamic polarization test, the scan position was initiated at 300 mV with a 0.167 mV/s scan rate and reversed when the potential reached 400 mV. Potentiostatic tests were conducted at room temperature (in 3.5 wt. % NaCl solution) to accelerate crevice corrosion.

### 2.4. Morphology Observation

A solution of 10% oxalic acid and 4% nitrate alcohol was used to etch SS and CS, respectively [22]. Optical microscopy (OM) and scanning electron microscopy (SEM, SU5000) with energy dispersive spectroscopy (EDS, Oxford, UK) were used to visualize the microstructures of the joints and corrosion morphology of SS cladding after polarization. The compositions on the cladding surfaces were determined by X-ray photoelectron spectroscopy (XPS), equipped with a monochromatic X-ray Al-Ka source with a pass energy of 25 eV. XPSPEAK41 software was used to process the data after calibrating the C1s peak at 284.8 eV. The corrosion profile and cross-section depth curves after removing the corrosion products were measured using a 3D ultra-depth microscope (CLSM, OLS5000-SAF, Olympus, Japan).

## 3. Results and Discussion

### 3.1. Microstructure of 304/45 SSCP Joints with Q-T Treatment

**Figure 2:**
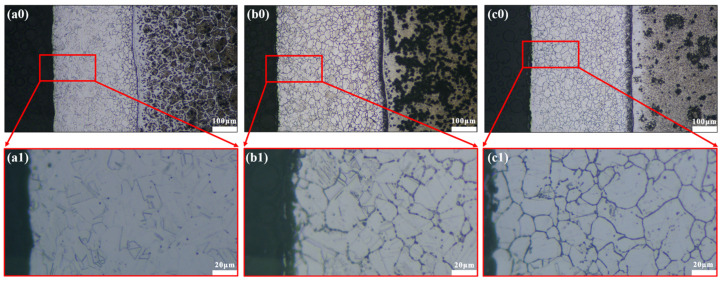
Microstructure of the SSCP with different Q-T treatment: (**a0**,**a1**) as-rolled, (**b0**,**b1**) QT500 and (**c0**,**c1**) QT600.

Seen from Figure 2a, the typical characteristics of 304/45 SSCP hot-rolling bonding joints showed a prominent carburized layer on the 304SS cladding and decarburized layer on the 45 steel side. The carburized layer thickened distinctly during the Q-T treatment in Figure 2a–c. Commonly, the diffusion of C along austenite intergranular leads to network precipitation. As a result, the SS cladding of specimens etched by oxalic acid electrolysis shows intergranular sensitization to a certain extent (Figure 2(a1–c1)). Compared with as-rolled SSCP, Q-T-treated SSCP had a significant change in the intercrystalline network distributed throughout the SS cladding. The Q-T treatment further promoted C diffusion from the CS side to SS side, and more C atoms that diffused into the SS cladding fully precipitated along the grain boundaries, while chromium carbide precipitated at the grain boundaries and even within the grains. Meanwhile, the quenching temperature and tempering temperature of the Q-T treatment are both within the range of the intergranular sensitization temperature of austenitic stainless steel, with more Cr_23_C_6_ precipitated in this temperature range (500–850 °C) [23]. Additionally, as the tempering temperature increased, the C diffusion and carbide precipitation behavior became accordingly significant.

Q-T treatment further promoted C diffusion from the CS side to SS side, with more C atoms that diffused into SS cladding fully precipitated along the grain boundaries, while chromium carbide precipitated at the grain boundaries and even within the grains.

**Figure 3:**
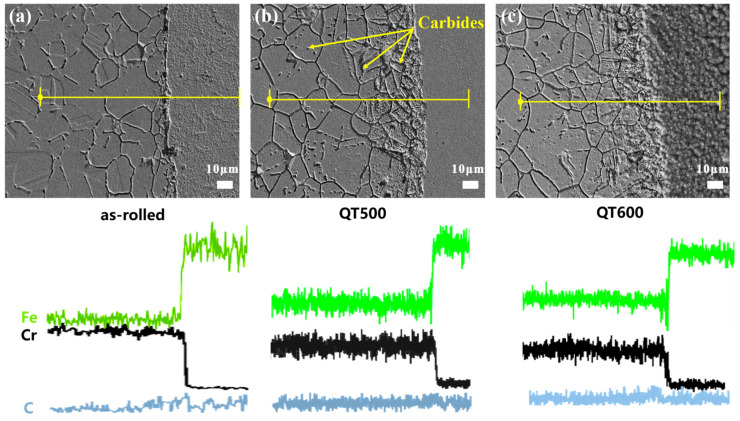
EDS line scanning of SSCP across the bonding interface with different Q-T treatments: (**a**) as-rolled, (**b**) QT500 and (**c**) QT600.

To better visualize the interfacial element diffusion across bonding joints, SEM was carried out, and the line scan results are shown in Figure 3a–c. Fe and Cr atoms noticeably interdiffused in the 304/45 joints. Moreover, the diffusion behavior of the C atom was not obvious [24], but there was a slight diffusion of C near the joints from the CS side to SS, as shown in Figure 3c. Both the austenite intergranular and intragranular carbides of SS cladding increased after Q-T treatment, but the growth trend at the boundary was more obvious than that in the grain. This phenomenon could be explained by the C atom preferentially diffused along the boundary in austenite grain [25]. Therefore, with the increase of the tempering temperature, the C atoms could rapidly diffuse to SS cladding, especially to the outer surface.

### 3.2. Electrochemical Behavior of SS Cladding

**Figure 4:**
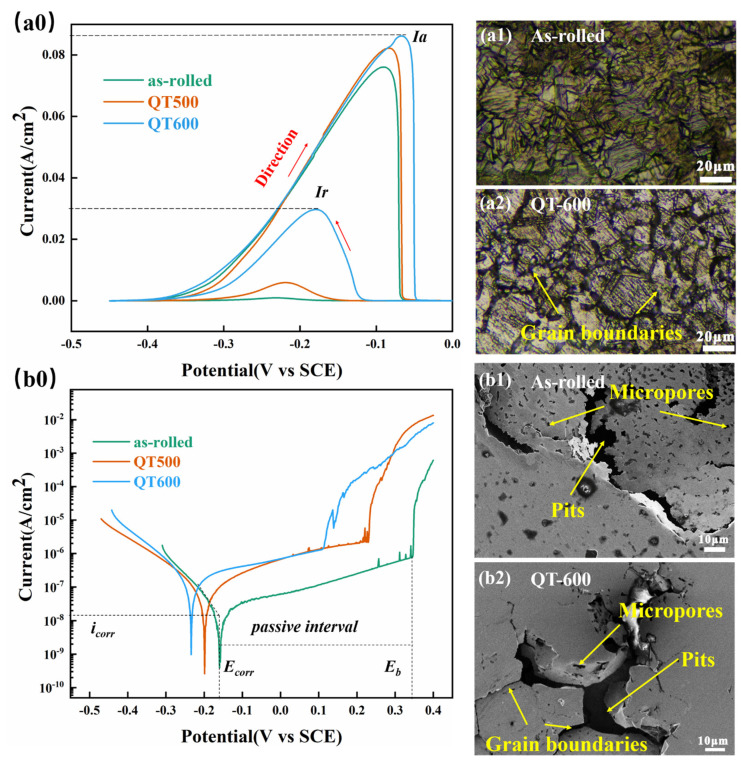
DL-EPR curve and corrosion morphology (**a0**–**a2**) and polarization curve and corrosion morphology (**b0**–**b2**) of SS cladding with different Q-T treatments.

For investigating the corrosion behavior of the outer surface of 304SS cladding on the SSCP, double-loop electrochemical potentiokinetic reactivation (DL-EPR) and polarization were employed to estimate the intergranular and pitting sensitization of SS cladding, respectively. Figure 4(a0) depicts DL-EPR curves obtained from SS cladding after Q-T with different tempering temperatures. The degree of sensitization (DOS), known as the ratio of *I_r_*/*I_a_*, for the specimens was detected according to the DL-EPR curves in Table 3. The value of the DOS raised from 1.2% (as rolled) to 34.5% (QT600) due mainly to the positive precipitation of chromium carbide when the tempering temperature increased. Notably, the variation of DOS was in keeping with the enrichment of Cr_23_C_6_. A higher reduction peak (*I_r_*) indicated more precarious passive film, which was attributed to the formation of intergranular carbide and lack of intragranular free-state Cr [26]. Specifically, during Cr_23_C_6_ generation, consumed Cr could not be supplemented from austenitic grain in time, thus forming a certain width of intergranular Cr-depleted zones. On the one hand, the lack of free-state Cr in grain impacts the integrity of the passive film on the surface [27,28]. In addition, potential differences exist between Cr-enriched and Cr-depleted zones, resulting in active–passive microelectronic couple cells [29], leading to the accelerated destruction of passive film on the cladding surface. The corrosion morphologies of the as-rolled and QT600 samples after DL-EPR testing were analyzed. As seen in Figure 4(a1,a2), obvious etching traces appeared at the grain boundary of the specimen after Q-T treatment, which was related to its high DOS value.

Figure 4(b0) and Table 3 present the polarization results of SSCP outer SS cladding with different tempering temperatures in 3.5 wt. % NaCl solution at room temperature. Compared with as-rolled SSCP, the *E_corr_* of QT500 shifted negatively to −0.2 V, and the *I_corr_* increased rapidly to 8.5 × 10^−8^ A/cm^2^. Meanwhile, as the tempering temperature increased, the *E_corr_* of QT600 became lower, and *I_corr_* was higher at 2.1 × 10^−7^ A/cm^2^. Furthermore, the *E_b_* was apparently degraded, and the passive interval was narrowed. It is indicated that the passive film of 304SS cladding was vulnerable to breaking down. In summary, the evolution of corrosion resistance of the cladding surface might be related to carbide precipitation during Q-T treatment. Figure 4(b1,b2) show the corrosion morphologies after polarization testing. Interestingly, pits appeared near the grain boundary, accompanied by the appearance of micropores. However, the as-rolled sample had a uniform distribution of micropores on its surface, but the QT600 sample had fewer micropores on its surface, which were replaced by further expanded intergranular corrosion pits. This may be due to the active site of Cr_23_C_6_ at the grain boundary, which provides a driving force for the attack of chloride ions.

### 3.3. Electrochemical Behavior of SS Cladding with PTFE Crevice-Former

**Figure 5:**
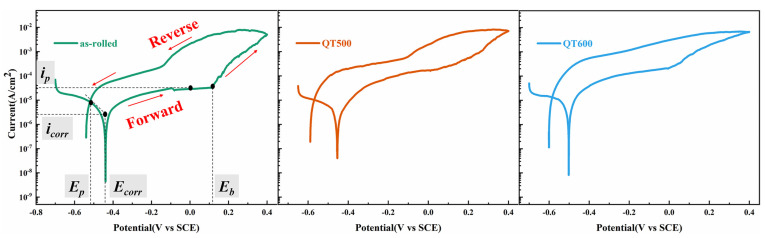
Cyclic potentiodynamic polarization curves of the SS gap with different Q-T treatments in 3.5 wt. % NaCl solution.

In this study, the crevice corrosion resistance of SSCP cladding was studied by cyclic potentiodynamic polarization. Figure 5 shows the curves of SS cladding with the PTFE crevice-former (SS gap) under different tempering temperatures in a 3.5 wt. % NaCl solution. The corrosion potential (*E_corr_*), breakdown potential (*E_b_*), re-passivation potential (*E_p_*), corrosion current density (*I_corr_*) and passivation current density (*I_p_*) at 0 V were acquired from Figure 5 and are exhibited in Table 4. Compared to the tests without the crevice-former in Figure 4b, the *I_corr_* and *I_cp_* increased distinctly, because localized anodic reactions inside the crevice accelerated the destruction of the passive film.

On the basis of previous studies, the *E_p_* is defined as the intersecting potential of the forward and reverse scan polarization curves [30], the value of which means the degree of re-passivate SS cladding. Additionally, crevice corrosion did not occur unless the *E_corr_* value of the metal in the corrosive environment exceeded the *E_p_* [31]_._ Below the *E_p_*, the crevice corrosion cannot spread, and the SS cladding surface began to re-passivate [32]. Comparing only the cyclic potentiodynamic polarization test of three specimens with Q-T treatment from Figure 5, all *E_p_* values of as-rolled (−0.52 V), QT500 (−0.57 V) and QT600 (−0.59 V) were lower than that of *E_corr_*. The specimen QT600 with the lowest *E_p_* values possessed the maximum risk of crevice corrosion. It can be seen that the QT600 specimen had the lowest *E_b_* value, proving the assembly crevice was more vulnerable to being attacked. After the breakdown, the current density increased remarkably, revealing the start and expansion of crevice corrosion [29]. When the potential sweep began to reverse, the current did not immediately decrease and remained at about 10^−2^ A/cm^2^ for a period of time. This indicates that the crevice corrosion continued to expand [33].

**Figure 6:**
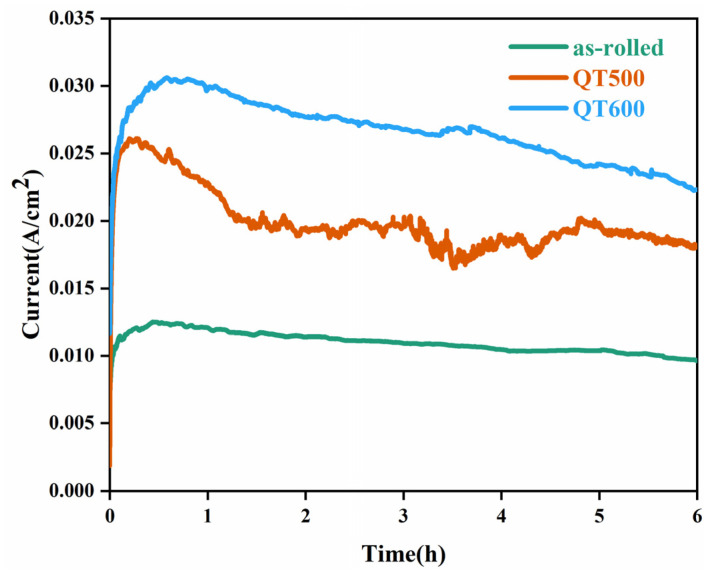
Potentiostatic polarization curves of the SS gap with different Q-T treatments in 3.5 wt. % NaCl solution for 6 h.

For investigating corrosion morphology differences on the SS cladding surface by accelerated tests, a 0.2 V constant voltage was applied to the SS gap for 6 h. Figure 6 depicts the potentiostatic polarization curves of crevice specimens with different Q-T treatments in 3.5 wt. % NaCl solution. It was noticed that the current density of the Q-T state was significantly greater than that of the hot-rolled state. In addition, the current density increased at the same voltage and time as the increasing tempering temperature. The corrosion current represents the severity of crevice corrosion, as corrosion becomes severe as the corrosion current increases.

### 3.4. Corrosion Morphology of Stainless Steel Cladding

**Figure 7:**
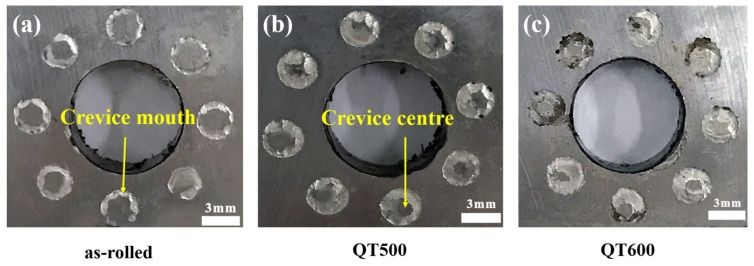
General corrosion morphology of SS cladding in 3.5 wt. % NaCl solution after potentiostatic polarization for 6 h with different Q-T treatments: (**a**) as-rolled, (**b**) QT500 and (**c**) QT600.

It could be found that all crevice regions of SS cladding suffered localized corrosion, the annular corrosion pit morphology shown in Figure 7, specificially due to the low compressive strength of PTFE crevice-former producing extrusion deformation when applying torque to fix the crevice device. As a result, the width of the crevice central part was less than 0.025 mm, which could not satisfy the requirement of the initial crevice corrosion [13]. What is more, the rate of crevice growth and solution migration to the interior was slow, because PTFE was corrosion-free in the NaCl solution.

**Figure 8:**
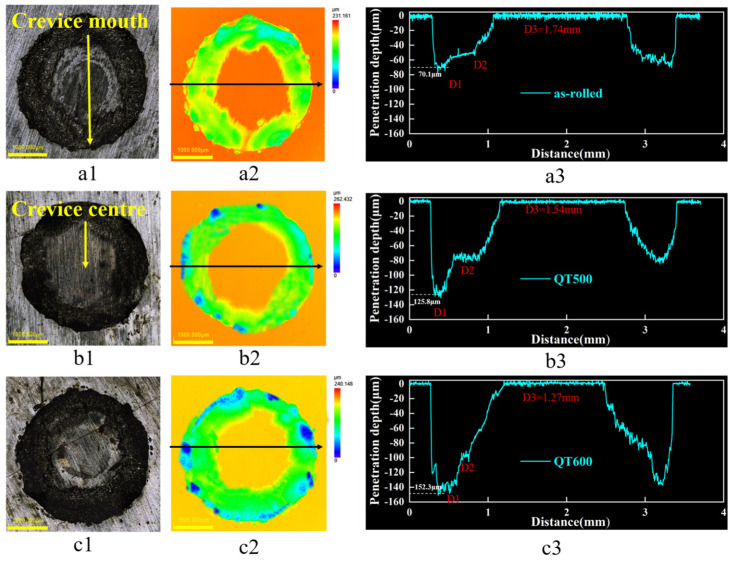
Micromorphology (**a1**,**b1**,**c1**), corrosion profile (**a2**,**b2**,**c2**) and corrosion depth (**a3**,**b3**,**c3**) of the SS gap in 3.5 wt. % NaCl solution after potentiostatic polarization for 6 h with different Q-T treatments: (**a**) as rolled, (**b**) QT500 and (**c**) QT600.

Figure 8 depicts the corrosion profile, including the width and depth of the SS gaps with Q-T treatment. The depth curve in Figure 8(a3,b3,c3) was obtained by scanning along the direction of the black arrow in Figure 8(a2,b2,c2). From cross-sectional morphology, the corrosion profiles of all specimens could be divided into three parts; these were D1, D2 and D3. All samples had suffered different degrees of corrosion after potentiostatic polarization, resulting in the most serious corrosion and the deepest morphology at the D1 area. Interestingly, a platform with a certain width at D2 in the specimens with Q-T treatment could be associated with the distribution differences of carbide on SS cladding. The D3 areas were found in the crevice-former center without being attacked by a corrosion medium. Consequently, the corrosion depth of D3 was near 0. As seen in Figure 8(a3), the D1 depth in the SS gaps of the as-rolled specimen was 70.1 μm. The corrosion pits extended from the D1 direction to D2 approximately parallel, where the depths of D1 and D2 were similar. However, the maximum corrosion depth in the SS gaps of QT500 was 125.8 μm at the D1 area. Compared to the as-rolled state, the overall corrosion pit depth increased, and the width of the platform D2 area decreases. It is worth noting from the SS gaps of QT600 in Figure 8(c3) that the maximum pit deepened to 152.3 μm when tempering temperature increased. D2 showed a deeper depth and appeared as a slope instead of platform morphology.

Surprisingly, the pit’s growth revealed the crevice corrosion behavior of SS gaps in specimens with different Q-T treatments. During the incubation stage of the corrosion pits, the crevice mouth was attacked and started being etched longitudinally as the priority. Subsequently, the pits were enlarged further in the deep-forming D1 area and prolonged horizontally in the width-forming D2 platform. Finally, SS cladding was prejudiced, bringing about deeper pits and an evanescent platform at the D2 area. All phenomena were closely related to the variation of the SS cladding microstructure after Q-T treatment with the tempering temperature.

**Figure 9:**
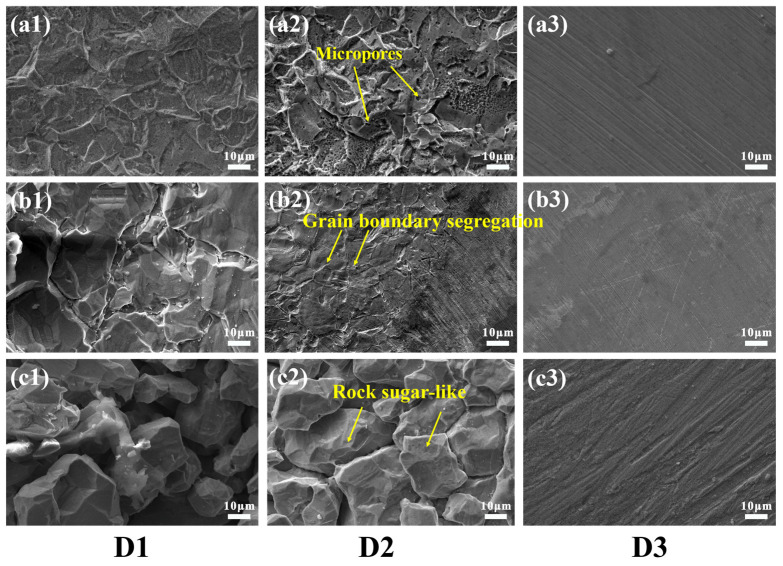
SEM micrographs of different regions of SS cladding in 3.5 wt. % NaCl solution after potentiostatic polarization for 6 h: (**a1**–**a3**) as rolled, (**b1**–**b3**) QT500 and (**c1**–**c3**) QT600.

For investigating the relevance between the cladding microstructure and its crevice corrosion, SEM micrographs of different regions in the SS gaps after electrochemical polarization are exhibited in Figure 9. This illustrates the magnified images at the crevice corrosion regions of 304/45. Numerous corroded micropores are exhibited uniformly at the D1 and D2 areas in Figure 9(a1,a2). This was similar to the corrosive pitting morphology of SS cladding surface after polarization. Figure 9(b1–b3) show the crevice corrosion morphology of the QT500 specimen. The grain boundary segregation of micropores was detected at the D2 platform, as seen in Figure 9(b2). However, the intergranular erosion dents at the D1 area were relatively deeper. The increasing intergranular sensitization after Q-T treatment led to Cr_23_C_6_ boundary segregation and being attacked preferentially. Meanwhile, the crevice pits grew downward with the rise of Cr_23_C_6_ formation near the SS/CS bonding interface. Nevertheless, more telling evidence is shown in Figure 9(c1–c3). Both the D1 and D2 areas had typical intergranular failure morphology that was rock sugar-like morphology in the SS gaps of the QT600 specimen after the crevice corrosion test. In addition, the content of Cr in the SS gaps of specimens with increasing tempering temperature evidently reduced from 14.65% (as-rolled) to 4.15% (QT600), and the content of C reduced slightly, shown in Table 5, which could prove the close relevance between SSCP crevice corrosion behavior and Cr_23_C_6_ particles generation, as mentioned before. Interestingly, corrosion-free areas could be found at the D3 regions in all crevice specimens. It is revealed that the intercrystalline corrosion of SS cladding on the SSCP surface after Q-T treatment might not be detonated spontaneously but could be initiated and accelerated by the existence of the crevice.

**Figure 10:**
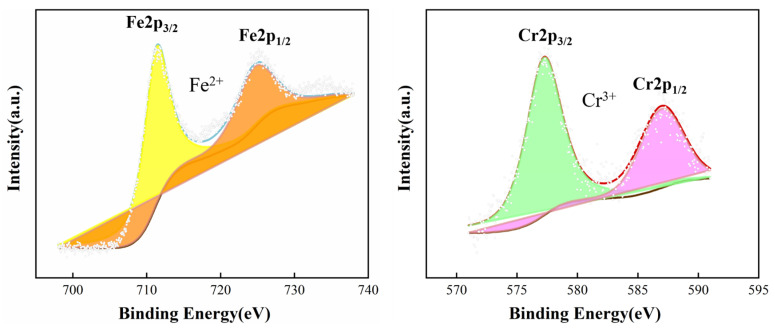
Measured XPS spectra of Fe2p and Cr2p of the QT600 specimen after crevice corrosion.

Notably, as seen in Table 5, the rise of O content indicated oxide reaction and generation. To gain a better understanding of these products, XPS of the SS gap after the crevice corrosion test is verified in Figure 10. It shows the spectra of Fe2p and Cr2p in the SS gaps of QT600 after crevice corrosion. For the Fe2p_3/2_ peak decomposition, the binding energy (BE) around 711.3 eV is the characteristic of Fe^2+^, and the BE around 724.5 eV is also the characteristic of Fe^2+^ for the Fe2p_1/2_ peak decomposition. The BE around 572.2 eV and 586.9 eV are the characteristics of Cr^3+^ for the Cr2p_3/2_ peak decomposition and Cr2p_1/2_ peak decomposition, respectively [34]. This indicated that the corrosion products had the presence of Fe(OH)_2_ and Cr(OH)_3_.

**Figure 11:**
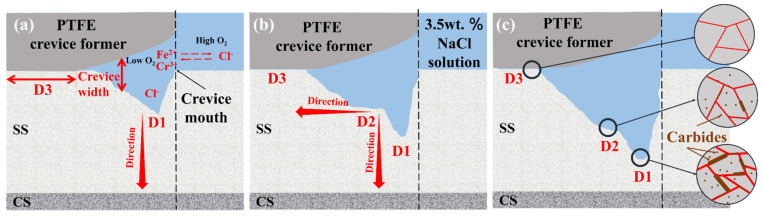
Schematic diagram of the crevice corrosion behavior of the SSCP in 3.5 wt. % NaCl solution: (**a**) initiation stage, (**b**) propagation stage and (**c**) development stage.

In summary, the processing of crevice corrosion could be divided into three parts, including the initiation stage, propagation stage and development stage. At the initiation stage of crevice corrosion for the SS gaps in the SSCP, Cl^−^ may invade the passive film on the SS cladding surface in 3.5 wt. % NaCl solution. It can be explained by the critical crevice solution theory [35]. The O_2_ inside crevice was consumed during long-term immersion and not available for immediate supplement. Subsequently, metallic ions were hydrolyzed, and consumed OH^−^ led to acidification inside the crevice. To maintain the electrical neutrality of the solution, cations and chloride ions migrated out of and into the crevice, respectively. This led to the breakdown of the passive film and the development of crevice corrosion, as shown in Figure 11a.

However, Cr_23_C_6_ precipitation in SS cladding of the SSCP after Q-T treatment became active sites for Cl^−^ attacking [36]. Corrosion was initiated at the crevice mouth of the SS gaps and thus extended to the inside of the cladding, i.e., the propagation stage. Subsequently, the corrosive pits could grow in two directions. For wider D2 direction, once corrosive pits formed, the distance between the crevice-former and SS cladding changed. As a result, the thicker compact center region was prolonged, and corrosive pits grew from the crevice mouth to crevice center. It is worth noting that the temperature during Q-T treatment provided a driving force for carbon atoms diffusion from the CS to SS side. Accordingly, Cr_23_C_6_ generation was gradient on the SS cladding, and its horizontal distribution density was similar. That is, the horizontal microstructure of SS cladding has the same inducing effect on the expansion of corrosion pits, which could be the key of the platform appearence at the D2 area. For the deeper D1 direction, these active sites further merged with the already-formed craters to form larger active sites, accelerating the degree of corrosion. Since the closer to the bonding joints, the more Cr_23_C_6_ precipitated, corrosion developed a priority for the bonding joints [37,38]. Therefore, Cr_23_C_6_ precipitated in SS cladding accelerated the development of crevice corrosion in the D1 direction. Distinctly, the crevice corrosion resistance of SS cladding on the SSCP could be weakened by the Q-T treatment, due to the excessive generation of Cr_23_C_6_.

## 4. Conclusions

The effects of Q-T treatment of the SSCP on crevice corrosion were discussed in a simulated seawater environment.

Q-T treatment promoted the diffusion of C atoms from the CS side to SS side on the SSCP. Accordingly, chromium carbide precipitated extensively at the grain boundaries and within grains, leading to severe intergranular sensitization. The corrosion resistance evolution of the SS cladding surface was directly related to the distribution density of chromium carbide in the SSCP. Q-T-treated SSCP showed lower re-passivation potential (−585 mV) when compared to as-rolled SSCP (−522 mV), with the maximum corrosion depth ranging from 70.1 μm to 150.2 μm. Corrosion morphology of the most severe area for as-rolled SSCP was numerous corroded micropores, while QT600 SSCP exhibited a typical intergranular corrosion morphology. The closer to the bonding interface, the more chromium carbide precipitated. Hence, corrosive pits developed along the grain boundary in a deeper direction. It greatly reduced the crevice corrosion resistance of the SSCP. The processing of crevice corrosion on SS cladding of the SSCP could be divided into three parts, including the initiation, propagation and development stages, which were quickened by the interaction of corrosive media and carbides. This work provides evidence for the study of element diffusion at joints of different bimetallic composite materials. Meanwhile, it plays an important role in the application of the SSCP in marine environments.

## Figures and Tables

**Table 1:** The chemical compositions of SS and CS (wt. %).

Materials	C	Si	Mn	P	S	Ni	Cr	Fe
SS (AISI 304)	0.055	0.44	1.21	0.028	0.003	8.13	18.28	bal.
CS (45 STEEL)	0.432	0.35	0.55	0.013	0.003	0.16	0.21	bal.

PS: balance (bal.).

**Table 2:** The schedules used in the heat treatment, along with the corresponding nomenclatures of the specimens.

Specimen Name	Bonding Parameter	Processing Conditions
as-rolled	hot rolling at 1150 °C for 1 h	Control group
QT500		Quenching at 850 °C for 0.5 h and WC + tempering at 500 °C for 1 h and AC
QT600		Quenching at 850 °C for 0.5 h and WC + tempering at 600 °C for 1 h and AC

PS: quenching and tempering (QT), water cooling (WC) and air cooling (AC).

**Table 3:** Electrochemical parameters of SS cladding with different Q-T treatments.

	*I_a_* (A/cm^2^)	*I_r_* (A/cm^2^)	DOS (*I_r_*/*I_a_* × 100%)	*E_corr_*/V	*I_corr_* (A/cm^2^)	*E_b_* (V)
as-rolled	7.6 × 10^−2^	9.35 × 10^−4^	0.012	−0.16	2.7 × 10^−8^	0.35
QT500	8.22 × 10^−2^	5.93 × 10^−3^	0.072	−0.20	8.5 × 10^−8^	0.23
QT600	8.61 × 10^−2^	2.97 × 10^−2^	0.345	−0.23	2.1 × 10^−7^	0.11

**Table 4:** Electrochemical parameters of the SS gap with different Q-T treatments.

	*E_corr_* (V)	*I_corr_* (A/cm^2^)	*I_p_* (A/cm^2^)	*E_b_* (V)	*E_p_* (V)
as-rolled	−0.44	1.6 × 10^−6^	2.9 × 10^−5^	0.11	−0.52
QT500	−0.45	5.7 × 10^−6^	1.7 × 10^−4^	0.025	−0.57
QT600	−0.50	1.1 × 10^−5^	2.2 × 10^−4^	−0.003	−0.59

**Table 5:** EDS element distribution of the D1 area after crevice corrosion: (**a**) as rolled, (**b**) QT500 and (**c**) QT600.

	(a) as-rolled (at%)	(b) QT500 (at%)	(c) QT600 (at%)
Fe	52.9	47.39	56.14
Cr	14.65	7.93	4.15
C	12.58	11.71	10.31
O	17.57	25.38	29.98

## Data Availability

Not applicable.
